# Perspectives surrounding fertility preservation and posthumous reproduction for adolescent and young adults with terminal cancer: Survey of allied health professionals

**DOI:** 10.1002/cam4.5345

**Published:** 2022-10-13

**Authors:** Francesca Barrett, Megan E. Sutter, Lisa Campo‐Engelstein, Amani Sampson, Arthur Caplan, Morgan Lawrence, Susan T. Vadaparampil, Gwendolyn P. Quinn

**Affiliations:** ^1^ Department of Obstetrics and Gynecology New York University Grossman School of Medicine New York New York USA; ^2^ University of Texas Medical Branch, Institute for Bioethics & Health Humanities Galveston Texas USA; ^3^ Division of Medical Ethics, Department of Population Health New York University Grossman School of Medicine New York New York USA; ^4^ Barnard College Columbia University New York New York USA; ^5^ Division of Population Science, Department of Health Outcomes and Behavior, Moffitt Cancer Center Tampa Florida USA

**Keywords:** adolescent and young adults, allied health professionals, fertility preservation, oncofertility, posthumous‐assisted reproduction

## Abstract

**Background:**

While all reproductive‐aged individuals with cancer should be offered fertility preservation (FP) counseling, there is little guidance over offers to adolescent and young adults (AYA) with terminal diagnoses, especially when considering posthumous assisted reproduction (PAR). The Enriching Communication skills for Health professionals in Oncofertility (ECHO/ENRICH) trains Allied Health Professionals (AHPs) to improve communication with AYAs with cancer. Little is known about AHPs' role in assisting in FP and PAR decisions.

**Methods:**

This is a cross‐sectional survey of ECHO/ENRICH trainees' attitudes and experience with FP and PAR in AYA with terminal cancer.

**Results:**

The response rate was 61% (365/601). While 69% felt comfortable discussing FP with terminal AYA after ECHO/ENRICH training, 85% desired further education. The majority (88%) agreed FP should be an option for AYA with cancer, though some agreed offering FP provided false hope (16%) or was a waste of resources (7%). Most shared that avoidance of FP discussions was common practice, especially in the medically fragile, late‐stage disease, or among minors. Many attributed lack of conversations to oncology team goals. Only 9% had prior experience with PAR. Many were conflicted about how PAR reproductive material should be gifted and who should be permitted to use PAR. Several raised moral concerns for PAR, or discomfort advising family. Many voiced desire for additional PAR‐specific education.

**Conclusion:**

ECHO/ENRICH trainees had varied levels of exposure to FP in terminal AYA and limited experiences with PAR. Many expressed uncertainties with PAR, which may be alleviated with further training and transparent institutional policies.

## Abstract 2

Allied Health Professionals who were ECHO/ENRICH trainees had varied levels of exposure to fertility preservation in terminally ill adolescent and young adults with cancer and limited experiences with posthumous assisted reproduction. Many expressed uncertainties with posthumous assisted reproduction, which may be alleviated with further training and transparent institutional policies.

## 1 INTRODUCTION

Adolescent and young adults (AYA) with cancer, defined as individuals aged 15 to 39 years at time of diagnosis, have unique psychosocial needs, with reproductive health being a chief concern.^1^, ^2^, ^3^, ^4^, ^5^ The American Society of Clinical Oncology (ASCO) guidelines suggest all patients of reproductive age diagnosed with cancer regardless of poor prognosis or late‐stage disease should be offered counsel, information regarding infertility and preservation options, and referrals to specialists for fertility preservation (FP); however, there is little guidance and great controversy over such offers to those with a terminal diagnosis.^6^ While there are ethical issues inherent to cancer and reproductive health among those with terminal diagnoses, none are more fraught with polarity than FP and the use of posthumous assisted reproduction (PAR).^7^, ^8^, ^9^

PAR encompasses the use of tissue, gametes, or embryos from deceased individuals for future family‐building attempts and includes cryopreservation prior to fertility‐compromising insult (gonadotoxic chemotherapy or surgery) as well as retrieval of tissue or gametes after death.^10^ In a national 2019 study among assisted reproduction clinics, 64% of clinics reported receiving requests for PAR in the past year, and 42% reported participating in PAR in the past 5 years.^11^ With this increased demand, the American Society of Reproductive Medicine (ASRM) Ethics Committee suggests that assisted reproductive clinics develop clear policies outlining the circumstances under which they would participate in PAR, and recommends that assisted reproductive clinic consent forms include specific directions regarding posthumous use of embryos and gametes, including specifying a bereavement period following the patient's death and prior to the use of the patient's gametes.^11^, ^12^ Despite these strong recommendations and the increasing requests for PAR services, many assisted reproductive clinics lack policies, and those with policies do not always follow ASRM recommendations.^10^, ^13^

Clinicians face unique ethical issues around discussions of fertility and preservation, which may be misinterpreted—from false hope regarding prognosis to encouragement of PAR.^10^, ^14^ The Enriching Communication skills for Health professionals in Oncofertility (ECHO) trains nurses, social workers, psychologists, and physician assistants (Allied Health Professionals [AHPs]) to improve communication about reproductive health with AYAs with cancer, to date training over 700 AHPs.^15^ Little is known about AHPs' awareness of PAR, institutional policies, and the role they may play in assisting patients, partners, or families in decision‐making about FP in the terminally ill and future PAR decisions. The purpose of this study was to assess AHP's awareness, perceptions, and experiences of FP among terminally ill AYA and PAR, and related institutional policies.

## 2 MATERIALS AND METHODS

### 2.1 Design

We conducted a cross‐sectional survey of 601 former ECHO/ENRICH learners between March 2020 and September 2020. This study was deemed exempt from Advarraa Institutional Review Board (IRB# 00000971).

### 2.2 Subjects

All AHPs who completed a prior ECHO course between 2011 and 2019 and for whom there was an accurate email address were eligible. Respondents were excluded if they did not complete at least 50% of survey items.

### 2.3 Survey development and distribution

**FIGURE 1:**
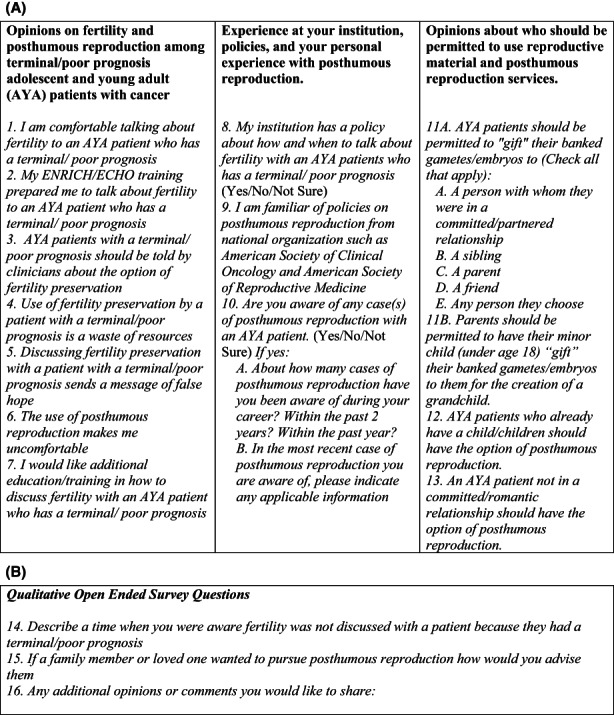
(A, B): Survey with Quantitative (A) and Qualitative (B) Attitudes of FP and PAR in terminally ill AYA with cancer. Applicable information in Figure 1A Question 10B included: Pre‐pubertal under 18 years, Post‐pubertal under 18 years, 18 years or older, Embryo cryopreservation, Oocyte cryopreservation, Sperm cryopreservation, Ovarian tissue cryopreservation, Testicular tissue cryopreservation, a partner, a sibling, parents, someone else, Not Sure/Do not Know.

A 16‐item survey was developed with 13 quantitative items and three open‐ended items on viewpoints related to FP and PAR (Figure 1A,B). The majority of quantitative items were taken from a national physicians' survey.^14^, ^15^ Items assessed attitudes toward FP among the terminally ill and the use of PAR; awareness of policies on FP and PAR; and personal experiences with cancer patients in the use of FP and PAR. Quantitative response options were on a Likert scale ranging from 1 = strongly agree to 5 = strongly disagree, or trichotomous questions with “Yes”, “No”, “Not Sure” options. Demographics were collected on profession, religious preference, role of religion in professional practice, race/ethnicity, gender, and sexual orientation. The survey link was electronically distributed via email using Qualtrics software. The Dillman method was used to remind respondents of the survey again at 3 weeks and 7 weeks after the initial link was sent.^16^ All participants were presented with an information sheet at the beginning of the survey and acknowledged consent to be a part of the study by advancing to the next page. All participants received a $10 gift card.

### 2.4 Data analysis

Prior to data analyses, quality control, value, and logic checks were performed to ensure data completeness and detect implausible values. Descriptive results are reported as counts or percentages. Categorical variables were analyzed using Chi‐squared or Fischer's exact test using SPSS Version 25 for Windows (IBM Corporation, Armonk, NY). An alpha error of 0.05 was considered statistically significant. Qualitative responses were analyzed using thematic content analysis. Open coding was applied to identify key themes within each question. Using an Excel file created from all responses and divided by question to which it pertained, three coders (FB, AS, ML) reviewed all responses and generated a list of potential codes, noting the strength of the pattern in the data. Codes were refined and reorganized into key themes and sub‐themes until consensus. Inter‐rater reliability was 92%, as assessed by Cohen's Kappa statistic. Subgroup analysis was additionally performed to evaluate for differences in demographic, professional, and religious factors that might impact AHP's perceptions.

## 3 RESULTS

### 3.1 Demographics

Sixty‐one percent (365/601) of ECHO/ENRICH learners responded to the survey, the analytic sample included 215 respondents who completed at least 50% of the survey. Of those, 164 respondents provided comments on the open‐ended questions. Most participants were cisgender, heterosexual women, identified with white race and/or non‐Hispanic/Latino ethnicity, and Christian (Table 1). Respondents were in practice a mean of 14.1 years (Std. 10.6 years).

**TABLE 1:** Demographic characteristics of former ECHO/ENRICH AHP respondents

	*n* = 215
Gender	
Cisgender woman	181 (84.2%)
Cisgender man	10 (4.7%)
Transgender woman	0 (0%)
Transgender man	0 (0%)
Non‐binary	0 (0%)
Other	2 (0.9%)
No response	22 (10.2%)
Ethnicity	
Hispanic/Latino	14 (6.5%)
Not Hispanic/Latino	184 (85.6%)
No response	17 (7.9%)
Race	
White	169 (78.6%)
Asian	11 (5.1%)
East Indian	1 (0.5%)
Native Hawaiian and Pacific Islander	1 (0.5%)
Black	8 (3.7%)
American Indian	2 (0.9%)
Multiple Races	3 (1.4%)
Other	1 (0.5%)
No response	19 (8.8%)
Sexual orientation	
Heterosexual	188 (87.4%)
Gay	2 (0.9%)
Bisexual	3 (1.4%)
Lesbian	1 (0.5%)
Pansexual	1 (0.5%)
Queer	2 (0.9%)
Not sure/questioning	2 (0.9%)
No response	17 (7.9%)
Religious identity	
Atheist or Agnostic	16 (7.4%)
Buddhist	4 (1.9%)
Christian (Any denomination)	129 (60%)
Hindu	2 (0.9%)
Jewish	12 (5.6%)
Muslim	0 (0%)
Not religious	27 (12.6%)
Other	9 (4.2%)
No response	16 (7.4%)
Licensure	
Registered nurse (RN)	86 (40%)
Advanced registered nurse practitioner (ARNP)	17 (7.9%)
Nurse practitioner (NP)	18 (8.4%)
Licensed clinical social worker (LCSW)	28 (13%)
Master of social work (MSW)	11 (5.1%)
Doctor of Philosophy (PhD)	17 (7.9%)
Doctor of Psychology (PsyD)	7 (3.3%)
Physician's assistant (PA)	16 (7.4%)
Other	17 (7.9%)
No response	2 (0.9%)
Years of practice	
<5 years	34 (13.5%)
5–10 years	62 (24.7%)
11–20 years	59 (23.5%)
21–30 years	23 (9.2%)
>31 years	24 (9.6%)
No response	49 (19.5%)

### 3.2 Opinions regarding FP and PAR in terminally ill AYA

**FIGURE 2:**
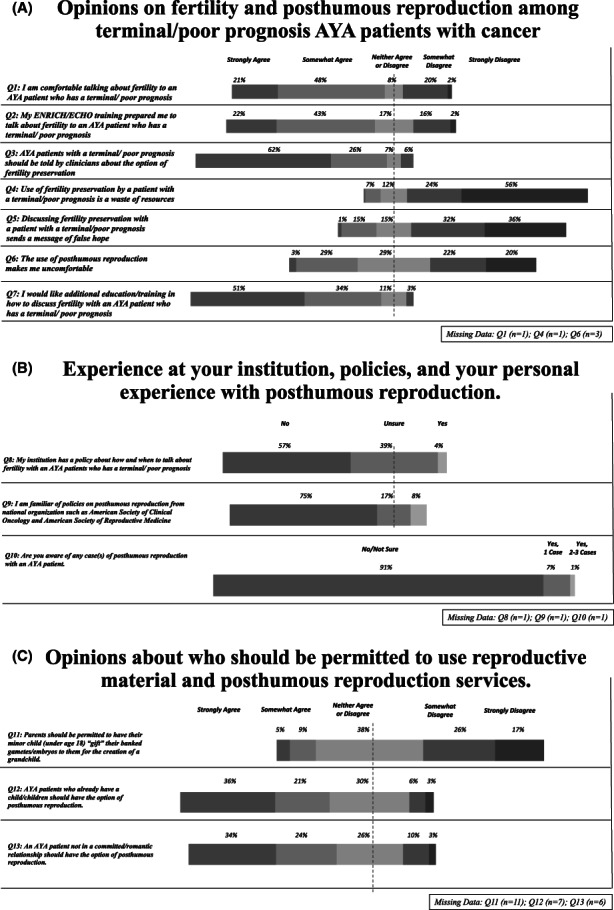
(A, B, C): Quantitative Results of Mix‐Method Survey Regarding Attitudes of FP and PAR in AYA with terminal cancer (A), Experiences of PAR (B), and Opinions on PAR Reproductive Material Gifting (C).

Opinions were largely favorable (strongly or somewhat agree) toward the use of FP in terminally ill AYA, though were divided regarded PAR (Figure 2A). Many participants (69%) felt comfortable discussing FP with terminal AYA patients and believed ECHO training prepared them for these discussions (65%). The majority desired further education (85%). Most agreed AYA patients with poor prognosis should be told about FP options (88%). Respondents primarily disagreed FP was a waste of resources for AYA (80%) and disagreed that discussions on FP sent messages of false hope (68%). On the topic of PAR, respondents were divided as to whether PAR made them uncomfortable: 32% agreed, 29% were ambivalent, and 42% disagreed.

Several demographic and professional characteristics were associated with responses to attitudes on AYA FP and PAR. Cisgender women were significantly more interested in receiving additional training with terminally ill AYA patients than cisgender men (*p* = 0.028). PhDs more strongly agreed with the need for additional training compared to their counterparts (*p* = 0.0099). Compared to other AHPs, nurse practitioners were more likely to agree that discussing FP may send a message of false hope (*p* = 0.008). Respondents who had been in practice for greater than 10 years more frequently disagreed that the option of PAR should be available for those who were not in a committed/romantic relationship (*p* = 0.082). Respondents who had been in practice for >20 years more often agreed that PAR was a waste of resources (*p* = 0.006).

There were no significant differences between how various religious groups viewed the impact of their religion in their practice or treatment of patients. Religion did play a role in opinions surrounding FP and PAR. Compared to all other religions, Christian participants were significantly more likely to agree that FP conversations send a message of false hope (*p* = 0.003) and are a waste of resources (*p* = 0.016) and to disagree that AYA patients who already had a child should have the option of PAR (*p* = 0.015).

### 3.3 Institutional experiences with FP and PAR

Most respondents were unfamiliar with specific policies surrounding the appropriate use of FP and PAR and few had had prior exposure to PAR (Figure 2B). Four percent of respondents were at institutions with specific policies surrounding how and when to discuss FP with terminal AYA, while 96% were unsure or unaware of their institution's policy. Eight percent were familiar with national organizational policies on PAR. The majority (91%) had no prior experience with PAR. Nine percent of participants were aware of at least one case of PAR with an AYA patient at their institution, of which 80% were in the past year and 95% were in the past 2 years. Most experiences involved cryopreservation of sperm (85%), with rare experiences involving testicular tissue (5%) or embryos (5%), and no experiences with ovarian tissue or oocytes. Posthumous gametes/embryos were used primarily by the partner (45%), parents (30%), a sibling (5%), or someone else (5%). Exposure to previous PAR cases was not associated with differences in awareness of institutional policies, though respondents were more likely to be aware of organizational policies surrounding PAR if they had encountered a personal PAR case (*p* = 0.002).

### 3.4 Opinions on gifting PAR reproductive material

Participants were conflicted about whom should be permitted to use PAR with many sharing ambivalent attitudes toward usage. The majority (88%) agreed AYA should be able to gift embryos/gametes to anyone, while 5% believed only to one's partner. Many agreed that patients who already had a child or those without a romantic relationship should have the option of PAR (57% and 54%, respectively). However, 43% disagreed that parents should be permitted to have their minor child “gift” them gametes/embryos for the creation of a grandchild.

### 3.5 Barriers to fertility discussions with terminal patients

The majority of respondents agreed that avoidance of FP discussions in AYA terminal patients was common practice (Table 2). Many participants attributed lack of fertility discussions to disease‐related factors, including terminal prognosis, late‐stage disease, medical fragility, emotional inability, lack of capacity, and concern for poor quality of life. Few additionally noted that conversations were avoided in minors or depending on one's relationship status.

**TABLE 2:** Sample representative quotes

Barriers to fertility discussions		
AHP specific barriers	Care team specific barriers	Protective factors
*“[avoiding conversations on fertility preservation] happens in nearly every case I can think of ‐ it is considered unnecessary.”*	“*…the doctor informed AYA team that it was not in patient's best interest to consider fertility because of his poor prognosis. It was difficult to initiate this conversation without the support of the medical provider. I am not even sure how clear the doctor was about the patient's prognosis”*	*Because of the class I had taken through ECHO, I was able to give them fertility preservation information and they were greatly [appreciative] for that*.
*“This is most of our patients given they have an 18–24 month prognosis. We only discuss fertility preservation in AYA patients that have a longer prognosis, unless they ask.”*	*“Medical team did not think it would be relevant or helpful for patient to hear about these options due to perception of it giving false home or extra worry”*	*“Additional training would be very helpful in terms of how to discuss FP and posthumous reproductive with patients and their families as well as how to educate medical providers that patients still have the right to FP even with poor prognosis.”*
*“This is not something I think to routinely discuss with patients with a terminal prognosis, particularly if they are young and/or not in a relationship.”*	*“…multiple times the phrase “your life is more important than your fertility” is used [by the medical team]. It is meant to relay if we cannot save your life what's the point.”*	
Advising a Loved one on PAR		
Practical responses related family and care team	Government/Policy responses	Ethical responses
*“I would have them see the fertility specialist to discuss their options in more detail. I would also have the social worker meet with them to discuss the emotional components related to this.”*	*“I would look at the laws/policies surrounding this issue at the federal/state level and also review what documents signed at the start of preservation from the sperm banking company regarding this matter.”*	*“… I would not say this out loud, but I would be concerned that they were doing it as part of the grieving process and as a way to “bring back” their loved ones.”*

Many found that the lack of fertility discussions was driven by the primary medical team (Table 2). Several highlighted insights into why the medical team avoided fertility conversations, ranging from discomfort surrounding the discussion, implicit bias regarding patient's desires, belief that FP was unnecessary, concern that gametes/embryos were unlikely to be utilized, or desire to focus on more pressing issues. Often, respondents noted that the oncology team's goals often were at odds with the patients. Few shared how the provider team was concerned for misleading the patient. Others shared how the medical team was primarily treatment focused rather than fertility focused for patients with poor prognoses.

Few respondents specifically cited the ECHO program as a way to overcome such barriers to have these discussions (Table 2). Respondents shared positive comments surrounding AYA PAR, ranging from gratitude, desire for further education on the topic, or expressing interesting in the area.

### 3.6 Advising a loved one on PAR

The majority of respondents answered with actionable and tangible ways they could advise their loved ones on PAR, including referring to experts, organizing family meetings, focusing on educational materials, or directing to institutional policies (Table 2). Most wished to direct their family members to specific providers, departments, or institutions. Most imagined referrals were to physicians, including oncologists, fertility specialists, and psychiatrists, though some planned to connect family members with social workers, ethics departments, or local fertility banks.

Several wanted to include the patient, family, loved ones in the conversation (Table 2). Several shared ways to provide emotional ways to support their family, such as exploration of values and motivation for PAR. Few advised that they connect with others who had been in the same situation or reach out to the ECHO team.

Few focused on rules and policies surrounding PAR, and furthering their own personal education on the matter (Table 2). Some raised moral and ethical concerns surrounding PAR, remarking on the complex ethical scenarios that could arise. Few noted that they could not be involved in their family's care. Few voiced concerns pertaining to PAR, such as fear for the impact of the future child or concerns related grief as a primary motivation for pursuing PAR. Several shared their personal beliefs surrounding PAR, voicing concerns surrounding the ethics, specific parameters for decision making, or expressing the complexity of these issues.

## 4 DISCUSSION

Counseling regarding the impact of cancer treatment on fertility and fertility preservation options is an important aspect of oncologic care, especially for AYAs with terminal cancer. AHPs play a vital role in the care of AYA cancer patients, often providing patients with the psychosocial care and support as well as health education related to complex decision making.^17^, ^18^ Our findings suggests that even among ECHO/ENRICH trained AHPs with the tools to discuss reproductive issues in cancer health, there are varied levels of exposure to FP in terminal AYA and limited experiences with PAR. Our study demonstrated several important findings regarding AHP's perspectives and experiences with FP and PAR in AYA with terminal cancer including: (i) the desire for additional education regarding FP and PAR (ii) the recognition of the importance of FP counseling with terminally ill AYA cancer patients (iii) the limited experience with institutional policies specific to FP and PAR (iv) the observed avoidance of FP discussions in terminal ill cancer AYAs, often driven by the primary medical team and (v) the discomfort or ambivalence of some AHPs with PAR discussions, gifting of reproductive materials, or ethics surrounding why one would pursue PAR. To our knowledge, this study is the largest cross‐sectional survey describing the experiences and perspectives of AHPs from across the United States on FP and PAR in AYA with terminal cancer.

Limited academic research exists surrounding PAR within the AYA population or regarding AHP's awareness, perceptions, and experiences of FP among the terminally ill and PAR, and related institutional policies. One prior study evaluating nurses' attitudes toward FP in patients with pediatric cancers revealed the limited conversations surrounding FP, concerns surrounding false hope, and difficulty finding FP resources for families, all of which are reinforced in our analysis.^19^ Another study on oncologic nurses revealed their perception that while FP counseling was important, it was outside their scope of practice, expressing lack of knowledge/comfort with the topic and expressing need for oncofertility education.^20^ In comparison, our ECHO cohort with explicit reproductive health training tools did not view FP discussions outside of their scope of practice but did desire further education to support their patients. Other studies have evaluated the role of the medical team regarding fertility preservation in AYA with terminal disease. Available studies suggest clinician discomfort in discussion of these topics because it may inadvertently give patients false hope regarding their prognosis.^7^, ^8^, ^9^ Oncologists appear uncertain on PAR and their views are significantly associated with their practice behaviors as well as their knowledge of ASCO guidelines.^14^ While we did not see a similar effect on knowledge of organizational guidelines, our respondents did highlight how the medical team often drove decision making and counseling surrounding FP and PAR. Finally, attitudes toward reproductive practices including assisted reproductive technology and PAR varied among Christian groups, with specific denominations disapproving of aspects due to implications of insemination of an unmarried woman or the idea of an embryo having a moral status as a human being.^19^, ^21^, ^22^, ^23^ As requests of PAR becomes more common, reproductive health providers will need further training and more transparent institutional policies to support the needs of terminal AYA patients.

Future policies should also give consideration to some of the ethical issues inherent in PAR. Lack of consideration of the principals of autonomy and justice would be a failure. For example, minors (aged 17 and younger) decisions on use of FP must include parents or guardians, though issues of discordance can arise which should be mediated with policies in place for mediation. Some ethicists have argued that non‐disclosure of the option for PAR violates patient autonomy.^24^, ^25^, ^26^ However, it is understandable that clinicians may not be comfortable offering an option that cannot be provided within their institution. If a patient/family needs to seek specialty reproductive services, these clinics may have their own policies that may or may not align with the cancer center. The potential for this should also be addressed in the policy which may alleviate some of the concerns expressed by the AHP respondents in this study regarding a need for transparency. The ethical issue of justice, fairness, equal, and equitable access also must be considered as part of policy.^27^ The financial costs for the use of FP and PAR vary by state as does insurance coverage.^28^ Access to specialty reproductive care may be hindered by the ability to travel to such care and the affordability in both time and resources to seek care.

There are several limitations to our study, particularly surrounding generalizability. Respondents were homogenous in terms of their demographics and practices, and 41% did not complete at least 50% of the survey. We were unable to adequately assess the impact of sexual orientation, ethnicity, and race as our cohort was predominately Caucasian cisgender heterosexual women. As well, as we did not collect the participants' current place of employment, some participants may be working at institutions that do not have a focus on fertility preservation and thereby do not need policies specific to PAR. The study population was based on involvement in an educational program specific to oncologic AHP. Therefore, our participants were likely more informed than those who had not had ECHO/ENRICH training and may have had more defined opinions regarding FP and PAR. Thus, the underlying representation of current participants to AHPs broadly is limited. As well, all results were self‐reported and prior experiences of communication surrounding FP or PAR were not objectively evaluated by clinical observation or chart review.

The high frequency of reported barriers to discussions surrounding FP and PAR in AYA patient with cancer with terminal prognosis remains a challenging clinical issue. Conversations surrounding FP can be valuable to patients, regardless of their prognosis.^8^, ^29^ Even among patients who do not pursue FP, it is unethical to limit discussions based on medical history or personal provider beliefs.^29^

In conclusion, ECHO/AHPs were unaware of FP policies and experienced limited conversations on FP and PAR in AYA patients with poor prognosis. Participants were uncertain on issues surrounding PAR and specifically the ethics surrounding gifting of gametes, though were interested in additional training on this topic. Respondents generally had positive attitudes toward the need for fertility counseling in AYA patients with cancer regardless of their prognosis, with few voicing concerns of false hope or wastefulness, though most encountered barriers due to the patient's medical status, treatment history, the medical team, or psychosocial factors preventing these conversations from occurring in their clinics. Further research is needed to evaluate the need for curricula specific to PAR and FP to AYA cancer patients with poor prognosis for AHP as well as other health care professionals. Additionally, further work is needed to support fertility clinics in creating transparent FP and PAR policies for AYA patients with terminal illness that are consistent with ASRM guidelines and to aid in the availability of this information to physicians, AHPs, and patients.

## AUTHOR CONTRIBUTIONS

**Francesca Barrett:** Formal analysis (lead); writing – original draft (lead); writing – review and editing (lead). **Megan Sutter:** Formal analysis (supporting); writing – review and editing (supporting). **Lisa Campo‐Engelstein:** Conceptualization (supporting); methodology (supporting); project administration (supporting); writing – review and editing (supporting). **Amani Sampson:** Conceptualization (supporting); formal analysis (supporting); methodology (supporting); writing – review and editing (supporting). **Arthur Caplan:** Conceptualization (supporting); methodology (supporting); writing – review and editing (supporting). **Morgan Lawrence:** Formal analysis (supporting). **Susan Vadaparampil:** Conceptualization (supporting); methodology (supporting); supervision (supporting); writing – review and editing (supporting). **Gwendolyn P Quinn:** Conceptualization (lead); supervision (lead).

## FUNDING INFORMATION

This study received NIH/NCI funding via the Bioethics Issues in AYA ECHO Administrative Supplement to R25: Enriching Communication Skills for Health Professionals in Oncofertility (NIH/NCI 3R25CA142520‐09S2).

## CONFLICT OF INTEREST

The authors confirm there is no personal or financial conflict of interest.

## Data Availability

Data sharing is not applicable to this article as no new data were created or analyzed in this study.
